# *Bartonella quintana* in Cynomolgus Monkey (*Macaca fascicularis*)

**DOI:** 10.3201/eid1112.030045

**Published:** 2005-12

**Authors:** Laurie G. O'Rourke, Christian Pitulle, Barbara C. Hegarty, Sharon Kraycirik, Karen A. Killary, Paul Grosenstein, James W. Brown, Edward B. Breitschwerdt

**Affiliations:** * University College Dublin, Dublin, Ireland; † North Carolina State University, Raleigh, North Carolina, USA; ‡ Novartis Pharmaceuticals Corporation, East Hanover, New Jersey, USA

**Keywords:** Bartonella quintana, Trench fever, Non-human reservoir, Cynomolgus monkey, Macaca fascicularis, Hemotropic organism, 16S ribosomal DNA, RNase P RNA 23S ribosomal DNA, 16-23S rDNA internally transcribed spacer region, gltA, dispatch

## Abstract

We identified a *Bartonella quintana* strain by polymerase chain reaction amplification, cloning, and sequencing of DNA extracted from lysed erythrocytes and cultured colonies grown from peripheral blood collected from a captive-bred cynomolgus monkey (*Macaca fascicularis*). This report describes naturally acquired *B. quintana* infection in a nonhuman primate.

*Bartonella quintana*, transmitted by the human body louse (*Pediculus humanis*), is the etiologic agent for trench fever. Although Mooser experimentally infected a rhesus monkey with *B. quintana* >50 years ago, we report the first naturally occurring infection with *B. quintana* in a nonhuman primate (*1*).

A young adult female cynomolgus monkey (*Macaca fascicularis*), born October 1, 1998, in a breeding facility in Vietnam, was shipped on February 28, 2001, to Covance Inc. (Alice, TX, USA), where she was quarantined and acclimated by the vendor. On April 30, 2001, the monkey was shipped to Laboratory Animal Services, Novartis Pharmaceuticals Corporation (East Hanover, NJ, USA) and held in quarantine until released on June 15, 2001, for study and assigned an identification number of 1505. Numerous procedures, treatments, and screening tests were conducted by the vendor during the monkey's quarantine in Texas and before its arrival in New Jersey. These included the following: 1) vaccination against hepatitis A (genus *Hepatovirus*) and measles (genus *Morbillivirus*); 2) serologic testing for cytomegalovirus (subfamily *Betaherpesvirinae*; positive), herpesvirus B (family *Herpesviridae*, negative), simian type D virus (simian retrovirus; SRV-1, -2, and -3; negative), simian immunodeficiency virus (SIV, genus *Lentivirus*; negative), simian T-lymphotropic virus (STLV, genus BLV-HTLV retroviruses; negative); 3) testing by polymerase chain reaction for SRV-1, -2, and -3 (negative); 4) Mantoux skin test for *Mycobacterium tuberculosis* (negative ×4); and 5) treatment for endoparasites with albendazole and avermectin, for ectoparasites with insecticide dust, and for *Plasmodium* spp. with chloroquine and primaquine. During the course of routine microscopic review of no. 1505's peripheral blood collected pretest (July 9, 2001) and stained with Wright stain (Hema-Tek 2000, Bayer Corporation, Wright Stain Pak, Curtin Matheson Scientific Inc., Houston, TX, USA; erythrocytic morphologic changes (moderate to marked stomatocytosis, punctate discoloration, or polychromatophilic aggregation) suggestive of a hemotropic parasite were observed (Figure 1). Malarial parasites were not observed. At the resolution of light microscopy (≈2 μ), basophilic particles were identified in association with erythrocyte membranes, with less well-defined, pale-blue inclusions seen within erythrocytes. Mean corpuscular volume was increased (82.2 fL). Blood from the same K-EDTA collection tube was transferred to the Electron Microscopy Laboratory (Novartis) for both transmission electron microscopy (TEM) and scanning electron microscopy (SEM) evaluation. Although intra- and extra-erythrocytic bacterial organisms were confirmed by TEM, and SEM identified numerous pits, the morphologic characteristics were not unique identifiers for *Bartonella* spp. (Figure 2). Since the sample was discarded after aliquots were taken for electron microscopy, a new K-EDTA blood sample was collected for culture from monkey no. 1505 and sent on ice by overnight delivery to the Intracellular Pathogens Laboratory, North Carolina State University College of Veterinary Medicine. Clinical observations during the study dosing period were unremarkable, and no unusual lesions were observed at necropsy or during histologic examination of selected tissues.

**Figure 1:**
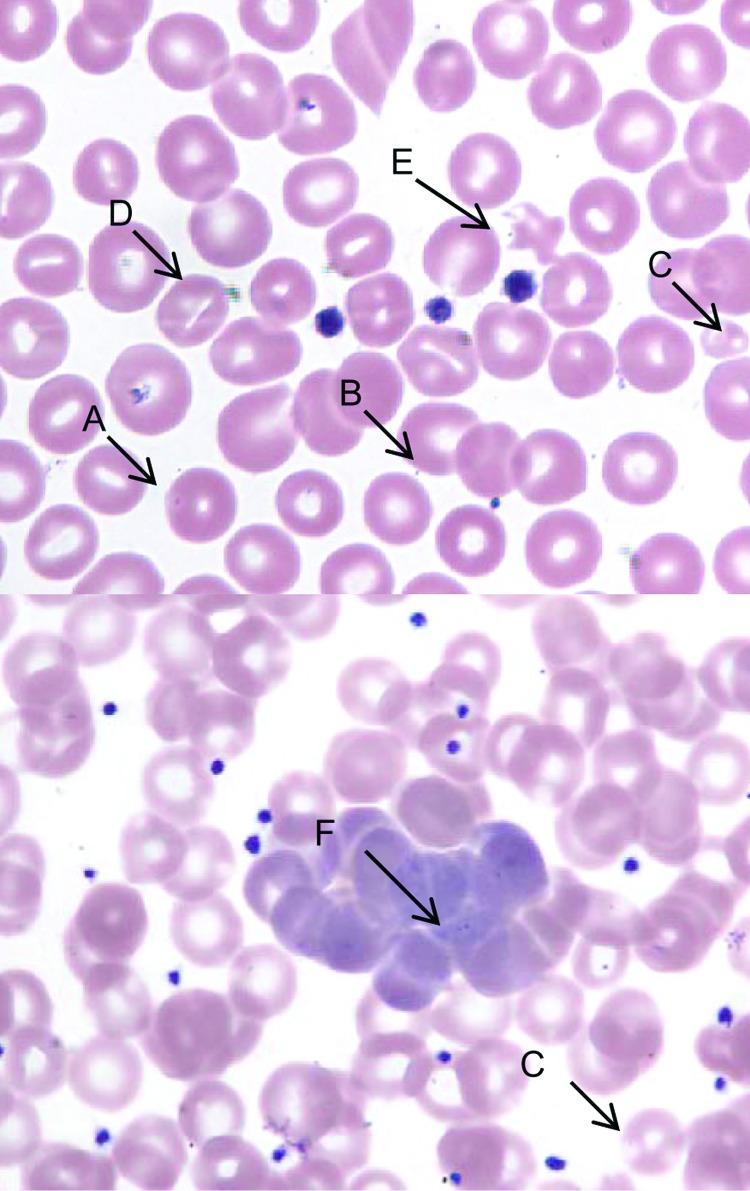
Peripheral blood film, Wright stain, 1,000× (oil immersion). A, spherostomatocyte with suspect intracellular organism; B, suspect membrane-associated organism; C, microcytes with punctate discoloration; D, stomatocyte; E, poikilocyte with punctate discoloration and suspected membrane associated organism; and F, aggregate of polychromatophilic erythrocytes and suspect intracellular organism at tip of arrow.

**Figure 2:**
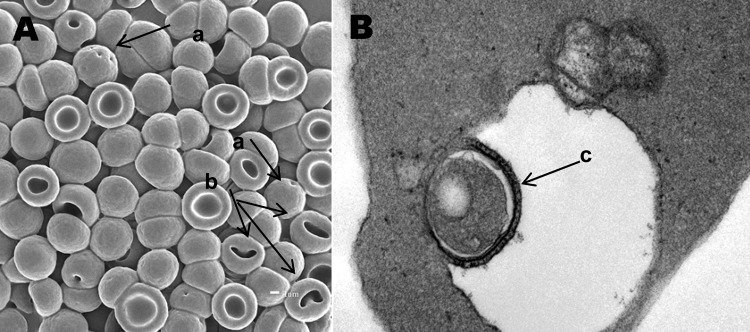
A) Electron microscopy scan of peripheral blood. B) Transmission electron microscopy scan of peripheral blood. a, membrane invaginations; b, stomatocytes and spherostomatocytes; c, erythrocyte with vacuole-enclosed suspect organism.

## The Study

Approximately 1.5 mL K-EDTA blood received from Novartis was frozen (–80°C) and then thawed 1 week later after lysis of the erythrocytes. After centrifugation of the sample at 3,000 × *g* for 30 min, the pellet was resuspended in M199 (Cellgro, Mediatech, Inc., Herndon, VA, USA) containing 20% (vol/vol) fetal bovine serum, 22.5% (vol/vol) sodium bicarbonate, 100 mmol/L sodium pyruvate and GlutaMAX-1 (Gibco Life Technologies, Grand Island, NY, USA) and spread onto trypticase soy agar containing 5% (vol/vol) rabbit blood and chocolate agar (Becton Dickinson, Cockeysville, MD, USA), respectively. Plates were incubated at 35°C under 5% CO_2_ and monitored for up to 6 weeks.

Five hundred microliters phosphate-buffered saline (PBS) was added to 200 μL blood (previously frozen at –80°C) and centrifuged at 20,817 × *g* for 6 min. The supernatant was removed, and the pellets were resuspended in 500 μL 1× PBS followed by centrifugation for 6 min. After removing the supernatant, and resuspending the samples in 200 μL PBS, we extracted DNA by using a QIAamp Blood Kit (Qiagen, Chatsworth, CA, USA) DNA from culture-grown *B. henselae* strain Houston-1, *B. vinsonii* subspecies *berkhoffii* (93CO-1), *B. elizabethae*, *B. clarridgeiae* (NCSU 94-F40), and *B. quintana* (ATCC VR-358) were used for all PCRs as control templates.

Amplification of the 16S rDNA and the 16S–23S intergenic spacer (ITS) regions was performed as described earlier (*2**,**3*). Amplification conditions for the citrate synthase gene (*gltA*) were the same as for the 16S–23S ITS region except that primers BhCS 1137n1 (5´ AATGCAAAAAGA ACAGTAAACA 3´) and CS443f 2 (5´ GCTATGTCTGCATTCTATCA 3´) were used (*4*). Selective PCR amplifications for the 16S rDNA, 23S rDNA, and *rnpB* were performed as described (*2*).

After cloning, recombinant plasmid DNA for *gltA* and the 16S–23S ITS region was sequenced bidirectionally with the infrared fluorescently labeled primers M13Reverse (5´ CAGGAAACAGCTATGACCATG) and T7 (5´ TAATACGACTCACTATAGGGCGA). The recombinant DNA carrying the genes for 23S rDNA, 16S rDNA, and *rnpB* was sequenced as described elsewhere (*5*). All sequences were aligned by using the multiple sequence alignment editor ALIGN-IR (LI-COR), and consensus sequences for every gene sequenced were determined. Consensus sequences were then used to identify the closest match within GenBank. To determine the exact phylogenetic relationship of the new isolate within the genus *Bartonella*, we analyzed an alignment that contained the sequences of 3 important phylogenetic markers, ribonuclease P RNA (RNase P RNA), 16S rDNA, and 23S rDNA, merged by catenation and organized by secondary structure elements, as described (*5*). Our dataset comprises 14 *Bartonella* strains (Table), including the 7 strains known to be human pathogens. We have also used the sequence information for the *gltA* as well as the 16S–23S rDNA ITS for sequence similarity analysis. Sequences have been deposited in GenBank with accession numbers AY484592 (16S rDNA), AY484593 (23S rDNA), AY484594 (RNase P RNA), and AY484595 (*gltA*).

**Table:** Strains used for the phylogenetic analysis within genus Bartonella*

Strain	16S rDNA	23S rDNA	*rnpB*
*B. vinsonii* subsp. *arupensis*^T^ (ATCC 700727)	AF214558	AF410937	AF441295
*B. clarridgeiae* strain NCSU 94-F40^T^ (ATCC 700095)	U64691	AF410938	AY033649
*B. doshiae* R18^T^ (ATCC 700133)	Z31351	AF410939	AF441294
*B. elizabethae^T^* (ATCC 49927)	L01260	AF410940	AY033770
*B. vinsonii* subsp. *berkhoffii* (93CO-1)^T^ (ATCC 51672)	L35052	AF410941	AF375873
*B. grahamii* V2 NCTC 12860^T^ (ATCC 700132)	Z31349	AF410942	AF441293
*B. henselae* strain Houston-1^T^ (ATCC 49882)	M73229	AF410943	AY033897
*Bartonella* strain N40	AF204274	AF410944	AF441292
*Bartonella* strain deer 159/660/1	AF373845	AF410945	AF376051
*B. quintana* strain Fuller ^T^ (ATCC VR-358)	M11927	AF410946	AY033948
*B. bovis* (formerly *B. weissii*) strain 99-BO1	AF291746	AF410947	AF376050
*B. vinsonii* subsp. *vinsonii* strain Baker ^T^ (ATCC VR-152)	Z31352	AF411589	AY033502
*B. bacilliformis* KC584	AF442955	L39095	AF440224
CMO-01-1	AY484592†	AY484593†	AY484594†

*T, type strains. GenBank accession numbers for sequences of the 16S rDNA, 23S rDNA, and *rnpB* are listed in corresponding columns.

†Accession numbers of sequence data determined in this study that were deposited into the GenBank database.

On day 14 after blood plating, growth typical for members of the genus *Bartonella* was obtained. Sixty-two small to medium-sized, white, shiny, smooth, nonadherent colonies were detected on chocolate agar. By day 16 after plating, 43 colonies of similar appearance were evident on blood agar. The strain was designated *Bartonella* strain CMO-01-1.

DNA could be successfully extracted, and subsequent PCR reactions resulted in PCR products representing 23S rRNA, 16S rRNA, RNase P RNA, 16S-23S rDNA ITS sequence, and the citrate synthase gene. All products were successfully cloned and sequenced. Sequencing of multiple clones for each gene resulted in sequences that were >99% identical to existing sequences derived from *B. quintana*, with the exception of the 16S–23S rDNA ITS sequence (>98.4%) and *gltA* (98%). Initial BLAST search results showed that the sequences for the 23S rDNA, the 16S rDNA, RNase P RNA, 16S–23S rDNA ITS sequence, and *gltA* derived from strain CMO-01-1 best matched *B. quintana* sequences that have been reported to GenBank. The data could be reproduced by using DNA extracted from the K-EDTA blood sample or from pure colonies grown on both chocolate and blood agar.

**Figure 3:**
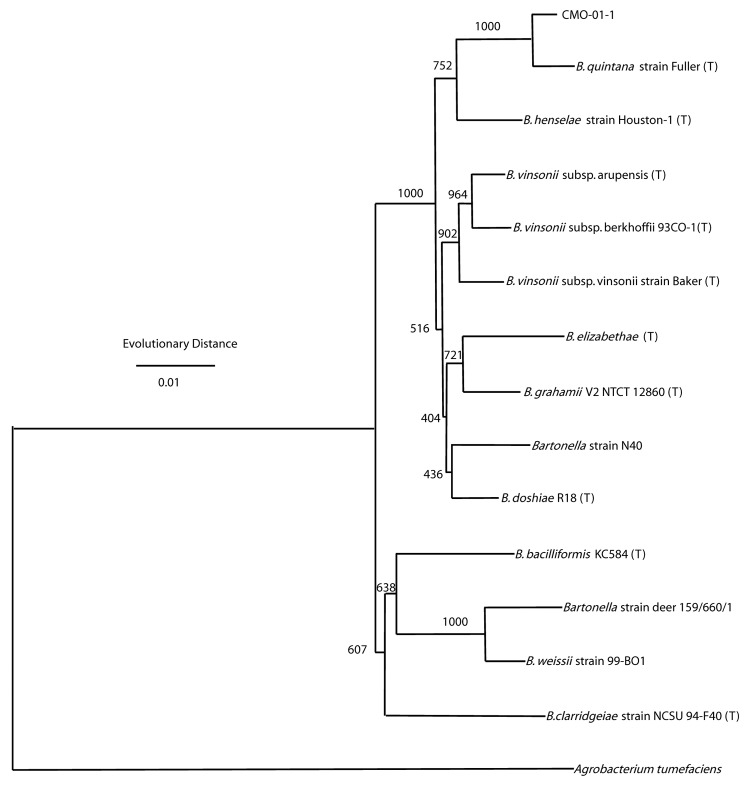
Phylogenetic tree of Bartonella species (Table) based on the combined RNase P RNA, 16S, and 23S rRNA sequence alignment. Agrobacterium tumefaciens serves as the outgroup in this tree. The tree shown was generated by using the neighbor-joining method. The horizontal axis is estimated evolutionary distance. The numbers shown at each node are the number of times that node appears among 1,000 bootstrapped trees. T, type strains.

Subsequent comprehensive phylogenetic analysis clearly identified the isolate CMO-01-1 as a close relative of *B. quintana* type strain "Fuller." The statistical support for this relationship is 100%, as indicated by the bootstrap values for the phylogenetic tree (Figure 3).

## Conclusions

Our findings support the close relationship of *B. quintana* and the new cynomolgus monkey isolate. The evolutionary distance (Figure 3) between *B. quintana* and the new isolate is similar to the evolutionary distance between the 3 subspecies of *B. vinsonii*. The high degree of sequence identity for the 16S rDNA of our isolate to other *B. quintana* 16S rDNA sequences deposited in GenBank clearly identifies strain CMO-01-1 as *B. quintana*. However, the high degree of 16S rDNA sequence identity makes a discriminatory match within or below the species level impossible. We have therefore applied the combined use of the phylogenetic markers RNase P RNA, 16S rRNA, and 23S rRNA for a comprehensive phylogenetic analysis. The advantage of such an analysis within the genus *Bartonella* has been discussed by Pitulle et al. (*2*). We have used the same dataset (Table) as described earlier (*2*) but added the sequences derived from the isolate CMO-01-1. We therefore consider the new isolate a novel strain of *B. quintana*.

The 1.6% sequence dissimilarities of the 16S–23S rDNA ITS data derived from CMO-01-1 to published 16S–23S rDNA ITS sequences for *B. quintana* are phylogenetically insignificant. The 16S–23S rDNA ITS region is a highly sequence variable area within the bacterial genome that can differ to the extent seen in our study at or below the species level (*3**,**5*). The sequences determined in our study have the same length as the 16S–23S rDNA ITS sequence reported for *B. quintana*, which further supports our conclusion that CMO-01-1 is a strain of *B. quintana*.

The *gltA* sequence derived from the bacterial strain CMO-01-1 is 98% identical to that derived from *B. quintana*. This match was the highest within the genus *Bartonella*. The next closest match was *B. henselae* with 92% similarity. This degree of sequence identity also suggests that CMO-01-1 represents a strain of *B. quintana*.

Confinement practices used for monkey 1505 should have eliminated or substantially restricted possible exposure to insect vectors, such as the human body louse (*Pediculus humanis*). Neither an exposure date nor an arthropod vector was identified in this monkey. Chronic subclinical infection with *B. quintana* as has been documented following experimental infection of monkeys (*1*) and in humans for *B. quintana* (*5**,**6*) and *B. bacilliformis* (*7*). Persistent infection was suspected but was not documented in this monkey because of the delay in bacterial identification. Bred and raised in outdoor facilities located in Southeast Asia, these primates often arrive in the United States with subclinical malaria, proof that exposure to mosquitoes and potentially other insects has occurred. *B. quintana* DNA has been recently found in ticks and fleas (*8**,**9*).

The reemergence of *B. quintana* infections in humans has expanded awareness of the organism's ability to induce persistent bacteremia in people with few symptoms (*5**,**6**,**10**,**11*). Because of the small number of infected erythrocytes needed to sustain infection, screening peripheral blood for organisms, even with confocal microscopy, has a poor success rate (*12*). Our findings indicate that nonhuman primates may serve as a previously unrecognized reservoir for human *B. quintana* infection.

## References

[1] Mooser H, Weyer F. Experimental infection of *Macacus rhesus* with *Rickettsia quintana* (trench fever). Proc Soc Exp Biol Med. 1953;83:699–701. 13120859 10.3181/00379727-83-20464

[2] Pitulle C, Strehse C, Brown JW, Breitschwerdt EB. Investigation of the phylogenetic relationships within the genus *Bartonella* based on comparative sequence analysis of the *rnpB* genes, 16S rDNA and 23S rDNA. Int J Syst Evol Microbiol. 2002;52:2075–80. 10.1099/ijs.0.02281-0 12508871

[3] Houpikian P, Raoult D. 16S/23S rRNA intergenic spacer regions for phylogenetic analysis, identification, and subtyping of *Bartonella* species. J Clin Microbiol. 2001;39:2768–78. 10.1128/JCM.39.8.2768-2778.2001 11473990 PMC88237

[4] Birtles RJ, Raoult D. Comparison of partial citrate synthase gene (*gltA*) sequences for phylogenetic analysis of *Bartonella* species. Int J Syst Bacteriol. 1996;46:891–7. 10.1099/00207713-46-4-891 8863415

[5] Brouqui P, Lascola B, Roux VR, Raoult D. Chronic *Bartonella quintana* bacteremia in homeless patients. N Engl J Med. 1999;340:184–9. 10.1056/NEJM199901213400303 9895398

[6] Foucault C, Barrou K, Brouqui P, Raoult D. *Bartonella quintana* bacteremia among homeless people. Clin Infect Dis. 2002;35:684–9. 10.1086/342065 12203165

[7] Kosek M, Lavarello R, Gilman RH, Delgado J, Manguina C, Verastegui M, Natural history of infection with *Bartonella bacilliformis* in a nonendemic population. J Infect Dis. 2000;182:865–72. 10.1086/315797 10950782

[8] Chang CC, Chomel BB, Kasten RW, Romano V, Tietze N. Molecular evidence of *Bartonella* spp. in questing adult *Ixodes pacificus* ticks in California. J Clin Microbiol. 2001;39:1221–6. 10.1128/JCM.39.4.1221-1226.2001 11283031 PMC87914

[9] Rolain J-M, Franc M, Davoust B, Raoult D. Molecular detection of *Bartonella quintana, B. koehlerae, B. henselae, B. clarridgeiae, Rickettsia felis*, and in cat fleas, France. Emerg Infect Dis. 2003;9:338–42. 12643829 10.3201/eid0903.020278 PMC2958535

[10] Brouqui P, Raoult D. *Bartonella quintana* invades and multiplies within endothelial cells in vitro and in vivo and forms intracellular blebs. Res Microbiol. 1996;147:719–31. 10.1016/S0923-2508(97)85119-4 9296106

[11] Spach DH, Kanter AS, Dougherty MJ, Larson AM, Coyle MB, Brenner DJ, *Bartonella* (*Rochalimaea*) *quintana* bacteremia in inner-city patients with chronic alcoholism. N Engl J Med. 1995;332:424–8. 10.1056/NEJM199502163320703 7529895

[12] Rolain JM, Foucault C, Guieu R, La Scola B, Brouqui P, Raoult D. *Bartonella quintana* in human erythrocytes. Lancet. 2002;360:226–8. 10.1016/S0140-6736(02)09462-X 12133660

